# Ability of shock index and heart rate to predict the percentage of body blood volume lost after vaginal delivery as an indicator of severity: results from a prospective cohort study

**DOI:** 10.7189/jogh.09.020432

**Published:** 2019-12

**Authors:** Anderson Borovac-Pinheiro, José Guilherme Cecatti, Rodolfo de Carvalho Pacagnella

**Affiliations:** Department of Obstetrics and Gynecology, School of Medical Sciences, University of Campinas, Campinas – Sao Paulo, Brazil

## Abstract

**Background:**

Postpartum hemorrhage (PPH) is the leading cause of maternal mortality worldwide, but it mainly affects women from low- and middle-income countries. Despite being a treatable condition, the high number of maternal deaths resulting from PPH is outstanding for at least 25 years. Late diagnosis and difficulties in identifying women who will develop severe postpartum bleeding can, in part, explain the high incidence of PPH. Over the past few years, researchers have focused on identifying a simple, accessible and low-cost diagnostic tool that could be applied to avoid maternal deaths. In particular, it has been suggested that vital signs and shock index (SI) could be useful. The objective of this study was to evaluate whether vital signs are correlated with the percentage of body blood volume (BBVp) lost after vaginal delivery.

**Methods:**

A prospective cohort study was performed at the Women’s Hospital of UNICAMP, Brazil. The inclusion criteria were women delivering vaginally who did not suffer from hypertension, hyper- or hypothyroidism, cardiac disease, infections or coagulopathy. Blood loss was measured over 24 hours using a calibrated drape and by weighing compresses, gauzes and pads. Vital signs were measured up to 24 hours after delivery. We evaluated the BBVp lost, and generated a Receiver operating characteristics (ROC) curve with area under the curve (AUC) analysis to determine the cut-off values for vital signs to determine the likelihood of postpartum bleeding above the 90^th^ percentile within 24 hours of delivery.

**Results:**

A total of 270 women were included. The mean blood loss within 24 hours of vaginal delivery was 570.66 ± 360.04 mL. In the first 40 minutes, 73% of the total blood loss over the 24-hour period had occurred, and within 2 hours, 91% of women had bled 90% of the total blood loss. Changes in SI and heart rate (HR) were statistically significant in predicting postpartum bleeding (*P* ≤ 0.05). Higher values for likelihood ratio (LR) to identify BBVp loss above the 90^th^ percentile within 2 hours were a SI above 1.04 at 41-60 minutes after birth (LR = +11.84) and a HR above 105.2 bpm at 21-40 minutes after birth (LR = +4.96). Both measures showed high specificity but low sensitivity.

**Conclusion:**

Values of SI and HR are statistically significant in predicting postpartum bleeding with high specificity but low sensitivity. The cut-off points were 1.04 for SI and 105 bpm for HR.

Postpartum hemorrhage (PPH) is the leading cause of maternal mortality, affecting approximately 10% of all women who give birth [1-4]. A reduction in global maternal mortality and morbidity is included in the Sustainable Development Goals, and the most powerful action to achieve this is by decreasing the incidence of PPH [3-5]. The World Health Organization (WHO) defines PPH as a visual estimation of blood loss of more than 500 mL within 24 hours of delivery [6]. However, some studies have reported that mean blood loss after delivery sometimes exceeds 500 mL, and this does not necessarily result in clinical complications [7]. On the other hand, some women who bleed less than 500 mL after childbirth will experience hemorrhagic shock [7-9]. These differences depend on individual characteristics like height, weight, presence of morbidities and the particularities of each gestation. Objectively, there are still doubts about the threshold amount of blood loss after childbirth associated with an increased risk of PPH.

The percentage of body blood volume (BBVp) lost after childbirth may play a role in distinguishing women who will experience clinical complications from those who will progress without disorders after losing the same amount of blood [10]. Body blood volume (BBV) is based on maternal body mass index (BMI), and therefore, it varies between different women [11]. The same absolute amount of blood lost after childbirth could mean higher BBVp loss for a woman with a low BMI and lower BBVp loss for a woman with a higher BMI.

Another factor that makes early identification of PPH more challenging is that diagnosis is based only on visual estimation of blood loss, as specified by the WHO. Therefore, it is of great interest to identify an effective complementary tool for identifying women who are at greater risk for developing severe maternal outcomes due to postpartum bleeding. In this way, changes in vital signs such as the shock index (SI) during the postpartum period have been studied as a simple, accessible and low-cost diagnostic instrument to help identify these women [8,12-14]. In low- and middle-income countries (LMICs), where the majority of hemorrhage-related maternal deaths occur, measurement of vital signs and SI is feasible and can support the decision to start treatment and/or transfer woman to a high-level facility before it is too late.

Considering the need to find a more accurate strategy to identify women who are at high risk of maternal death due to PPH, mainly in LMICs, the objective of this study was to prospectively identify changes in vital signs in postpartum women that are related to the percentile of BBV lost after delivery.

## METHODS

We conducted a prospective cohort study at the Women’s Hospital of the University of Campinas, Brazil, between February 1, 2015 and March 31, 2016. Recruitment of participants occurred at the time of admission to the hospital. Women were invited to participate and, if they accepted, they were asked to sign a consent form. The inclusion criterion was women who underwent vaginal delivery. The exclusion criteria were a gestational age below 34 weeks or the presence of any of the following pathologic conditions that could alter autonomic regulation: hypertension, hyper- or hypothyroidism (untreated), any cardiac disease, infections with fever or sepsis, and a history of coagulopathy.

Immediately after delivery, a calibrated drape was placed under each woman’s buttocks to measure blood loss (Brass-V Calibrated Obstetric Drape; Maternova Inc. Providence, USA). We measured blood loss at 5-minute intervals while the woman was in the birth position (including, for instance, while lacerations were sutured). Blood loss was calculated by summing the amount of blood in the drape to the weight of compresses and gazes used during the procedure (subtracting the dry weight). After this period, now without the calibrated drape, we measured blood loss every 15 minutes within 2 hours after delivery (4th stage of delivery) and from 2 to 24 hours after delivery, at every moment that the women discarded sanitary pads. All of the sanitary pads or drapes were collected in a sealed plastic bag for subsequent weighing. We calculated blood loss from the weight of sanitary pads (subtracting the dry weight). We used the blood density (1 g/mL) to calculate blood loss from the blood weight. [15] Every time blood loss was measured, the patient’s vital signs, including heart rate (HR), systolic (SBP) and diastolic blood pressure (DBP), respiratory rate (RR) and oxygen saturation (SatO_2_) were evaluated using a multiparameter meter.

We compared hemoglobin and hematocrit levels 24 hours after delivery with levels found in the antenatal records to calculate the drop-in hemoglobin due to postpartum bleeding. To decrease the influence of confounders, such as variation in hemoglobin levels during pregnancy, if the hemoglobin and hematocrit values in the antenatal records had been measured more than 3 months from the date of recruitment, we conducted a new hemoglobin and hematocrit exam.

The SI was calculated by dividing HR by SBP. The BBV was calculated according to BMI, and 95 and 73 mL/kg were used to assess BBV if the BMI was <35 or ≥35, respectively [11] The exact cut-off value for BBVp considered acceptable to lose after delivery has not been defined. Therefore, similar to other clinical definitions in medicine (eg, macrosomia and growth curves), we characterized bleeding above the 90^th^ percentile as abnormal. This assumption is also consistent with the prevalence of PPH, which occurs in around 10% of all deliveries. According to this prevalence, 90% of women will have normal bleeding after delivery, and the 10% with higher postpartum bleeding could potentially have PPH.

Exploratory data analysis was performed to determine the mean values, standard deviation, percentages and percentiles. To determine the association between mean BBVp lost and hemoglobin (Hb) decrease, we used the Mann-Whitney test. We calculated the 90^th^ percentile (p90) for postpartum bleeding within 2 and 24 hours, and identified the BBVp for specific time intervals. The mean SI and HR values observed for the following intervals were used in the analysis: 0-20 minutes, 21-40 minutes, 41-60 minutes, 61-90 minutes, and 91-120 minutes. For each interval, we evaluated the BBVp and used a receiver operator characteristic curve (ROC) analysis to determine the best cut-off point for vital signs to determine postpartum bleeding above the 90^th^ percentile at 2 and 24 hours after delivery. The areas under the curve (AUC) with the threshold values of SI and HR, sensitivity, specificity and positive and negative likelihood ratios were then calculated. A generalized estimating equations (GEE) model, with transformed blood loss in stations, was used to evaluate the ratio of SI to the BBVp over time. The level of significance was set at 5%, and SAS 9.4 software was used for all analyses.

This study was approved by the institutional Ethics Review Board (approval number 26787114.3.0000.5404). Women were provided with information about the study before going into labor and were invited to participate. If they accepted, they signed an informed consent form. If at some point during the data collection a woman no longer wanted to participate, she was excluded from the study.

## RESULTS

A total of 270 women were included in the study. Their sociodemographic and obstetric characteristics, in addition to measurements of blood loss, are shown in **Table 1**. The distribution of BBVp loss after childbirth is shown in **Table 2**. Women who bled 13.21% of their BBV or more in the first 2 hours after birth and those who bled 15.71% of their BBV or more within 24 hours of birth were classified as being in the 90^th^ percentile or higher.

**Table 1:** Obstetric and sociodemographic characteristics and measurements of blood loss among women after vaginal delivery

Characteristics	n	Mean ± SD
Age (in years)	270	24.67 ± 6.19
BMI* (In KG/m2)	244	28.85 ± 4.61
Parity	270	0.80 ± 1.10
Gestational age (in weeks)	270	38.93 ± 1.47
Mean percentage of BBV bled in 2 h†	266	6.06 ± 4.61
Mean percentage of BBV bled in 24 h‡	260	8.15 ± 4.99
Initial Hb‡ (in g/dL)	260	11.45 ± 0.10
Final Hb§ (in g/dL)	263	10.6 ± 1.58
Hb variation among patients who bled ≥20% of BBV in 2 h (in g/dL)	4	-1.32 ± 0.68
Hb variation among patients who bled ≥20% of BBV in 24 h (in g/dL)	6	-1.98 ± 1.15
**Ethnicity:**§		
White	178 (67%)	
Non-white	85 (33%)	
**Onset of labor:**		
Spontaneous	203 (75.19%)	
Induced	67 (24.81%)	
**Anesthesia/analgesia:**		
No	100 (37%)	
Yes	170 (63%)	
**Mode of delivery:**		
Vaginal	247 (91.5%)	
Forceps	23 (8.5%)	
**Episiotomy:**		
No	174 (64%)	
Yes	96 (36%)	
**Blood loss within 120 min of birth:**		
≥500 mL	84 (31%)	
≥1000 mL	22 (8.2%)	
**Blood loss within 24 h of birth:**		
≥500 mL	120 (44.5%)	
≥1000 mL	35 (12.9%)	

BBV – body blood volume, BMI – body mass index, h – hours, Hb – hemoglobin, SD – standard deviation

*Patient data missing from 26 patients.

†Patient data missing from 4 patients.

‡Patient data missing from 10 patients.

§Patient data missing from 7 patients.

**Table 2:** Percentiles of body blood volume percentage (BBVp) loss after childbirth (n = 270)

Period	5^th^	10^th^	25^th^	50^th^	75^th^	90^th^	95^th^
BBVp loss in 2 h	1.06	1.43	2.71	4.79	8.26	13.21	15.55
BBVp loss in 24 h	2.46	3.00	4.36	6.69	11.23	15.71	17.77

h – hours

The mean blood loss at 120 minutes was 427.49 ± 335.57 mL, while the mean blood loss at 24 hours was 570.66 ± 360.04 mL. Within 40 minutes, women bled 73% of the total blood lost in 24 hours, and within 2 hours, 91% of women bled 90% of the total blood lost. Among those who bled more than 1000 mL within 2 hours (n = 21, 8.1%) and within 24 hours (n = 35, 12.9%), the mean percentage of BBV lost was 14.89 ± 3.46% and 16.73 ± 2.81%, respectively. Twenty-seven women bled more than p90 within 2 hours. All participants bled more than 50 mL, and 20 (74%) bled more than 1000 mL. On the other hand, 243 women bled less than p90 and, among them, 56 (23%) bled more than 500 mL. Ten women refused the collection of blood after 2 hours following childbirth. Considering the amount of bleeding within 24 hours, 31 women bled above p90. All of the participants bled more than 500 mL and 27 (87%) bled more than 1000 mL. On the other hand, 87 (38%) women with bleeding below p90 bled more than 500 mL. Only four women bled more than 20% of their BBV within 2 hours after delivery. For these women, the mean blood volume lost was 1368.75 ± 210.01 mL, with a mean hemoglobin variation of -1.32 ± 0.68 g/dL. Within 24 hours of delivery, six women bled more than 20% of their BBV. For these women, the mean volume of blood lost was 1605.00 ± 291.34 mL, and the hemoglobin variation was -1.98 ± 1.15 g/dL.

Only four women required a blood transfusion. The mean blood loss, mean BBVp and hemoglobin and hematocrit variation for these women are presented in **Table 3**. There were no ICU admissions. Eleven patients received clinical treatment (additional oxytocin, methylergometrine or misoprostol), and only two needed surgical management (curation and curettage).

**Table 3:** Mean body blood volume (BBV) loss among transfused women (n = 4) at 2 and 24 h postpartum

	Mean ± SD
Blood loss within 2 h (in mL)	896.25 ± 651.59
Blood loss within 24 h (in mL)	1042.50 ± 702.98
Percentage of BBV loss within 2 h	11.70 ± 7.58
Percentage of BBV loss within 24 h	13.52 ± 8.00
Hemoglobin variation (in g/dL)	-2.65 ± 1.98
Hematocrit variation (in g/dL)	-8.07 ± 6.52

h – hours, SD – standard deviation

There were no statistical differences among SBP, DBP, RR and SatO_2_ in their ability to predict postpartum bleeding (*P* ≥ 0.5). There were, however, significant differences for SI and HR (**Table 4**).

**Table 4:** Correlation between vital signs and postpartum hemorrhage

	SI		HR		SBP		DBP		SatO_2_		RR	
**Period**	**Correlation**	***P-*value**	**Correlation**	***P-*value**	**Correlation**	***P-*value**	**Correlation**	***P-*value**	**Correlation**	***P-*value**	**Correlation**	***P-*value**
0-20 min	0.1814	0.0032	0.19409	0.0016	-0.00835	0.8934	0.02526	0.6852	0.11337	0.0696	-0.21599	0.0918
21-40 min	0.2209	0.0003	0.31043	<0.0001	-0.07713	0.2125	-0.02205	0.7219	0.04078	0.5118	-0.14637	0.2102
41-60 min	0.2194	0.0003	0.26496	<0.0001	-0.05704	0.3532	-0.02129	0.7292	0.14774	0.0161	-0.03028	0.7661
61-90 min	0.2581	<0.0001	0.30499	<0.0001	-0.03550	0.5636	0.06459	0.2930	0.11716	0.0578	-0.04430	0.6443
91-120 min	0.3229	<0.0001	0.43652	<0.0001	0.09574	0.2407	0.10913	0.1808	0.13122	0.1196	-0.04741	0.7099

SI – shock index, HR – heart rate, SPB – systolic blood pressure, DPB – diastolic blood pressure, SatO_2_ – oxygen saturation, RR – respiratory rate

**Table 5** shows the performance of SI and HR for identifying postpartum bleeding above the 90^th^ percentile at 2 and 24 hours (representing 13% and 15% of BBV, respectively). In the first hour after delivery, a SI above 1.0 has 97.5% specificity to identify a percentage of BBVp loss above the 90^th^ percentile at 2 hours after birth, and 93.4% to identify a percentage of BBV loss above the 90^th^ percentile at 24 hours. For HR, the best cut-off points for 2 and 24 hours were found to be 105 and 99 bpm, respectively, with 90.3% and 86% specificity. For SI, higher likelihood ratios (LRs) were found at 41-60 minutes, which were +11.84 and +4.89 for postpartum bleeding above the 90^th^ percentile over 2 and 24 hours, respectively. As for HR, higher LRs were found at 21-40 minutes after birth, which were +4.96 and +3.56 for postpartum bleeding above the 90^th^ percentile over 2 and 24 hours, respectively.

**Table 5:** Predictive ability of shock index and heart rate to identify postpartum bleeding above the 90^th^ percentile over 2 hours (13% of body blood volume) and 24 h (15% of body blood volume)

Blood loss	Period	AUC	*P-*value	Cut-off point	Sensitivity (95% CI)	Specificity (95% CI)	Likelihood ratio*	
**Shock index:**								
13% in 2 h	0-20 min	0.608	0.066					
13% in 2 h	21-40 min	0.675	0.003	0.80	59.3 (53.4, 65.2)	74.6 (69.3, 79.9)	+ 2.33 / - 0.55	
13% in 2 h	41-60 min	0.651	0.010	1.04	29.6 (24.1, 35.1)	97.5 (95.6, 99.4)	+ 11.84 / - 0.72	
13% in 2 h	61-90 min	0.705	<0.0001	0.83	55.6 (49.6, 61.6)	79.8 (75.0, 84.6)	+ 2.75 / - 0.56	
13% in 2 h	91-120 min	0.710	<0.0001	0.76	70.4 (64.9, 75.9)	69.8 (64.2, 75.4)	+ 2.33 / - 0.42	
**Heart rate:**								
13% in 2 h	0-20 min	0.662	0.006	102.1	63.0 (57.2, 68.8)	69.9 (64.4, 75.4)	+ 2.09 / - 0.53	
13% in 2 h	21-40 min	0.693	0.001	105.17	48.1 (42.1, 54.1)	90.3 (86.7, 93.9)	+ 4.96 / - 0.57	
13% in 2 h	41-60 min	0.687	0.001	99.50	48.1 42.1, 54.1)	84.9 (80.6, 89.2)	+ 3.19 / - 0.61	
13% in 2 h	61-90 min	0.675	0.003	94.75	51.9 (45.9, 57.9)	81.1 (76.4, 85.8)	+ 2.75 / - 0.59	
13% in 2 h	91-120 min	0.692	0.001	93.17	63.0 (57.2, 68.8)	76.6 (71.5, 81.7)	+ 2.69 / - 0.48	
**Shock index:**								
15% in 24 h	0-20 min	0.589	0.106					
15% in 24 h	21-40 min	0.637	0.015	0.80	53.3 (47.2, 59.4)	74.4 (69.1, 79.7)	+ 2.08 / - 0.63	
15% in 24 h	41-60 min	0.630	0.019	0.96	32.3 (26.6, 38.0)	93.4 (90.4, 96.4)	+ 4.89 / - 0.72	
15% in 24 h	61-90 min	0.652	0.006	0.71	74.2 (68.9, 79.5)	53.9 (47.8, 60.0)	+ 1.61 / - 0.48	
15% in 24 h	91-120 min	0.721	<0.0001	0.75	74.2 (68.8, 79.6)	68.4 (62.7, 74.1)	+ 2.35 / - 0.38	
**Heart rate:**								
15% in 24 h	0-20 min	0.664	0.003	102.1	67.7 (62.0, 73.4)	71.6 (66.1, 77.1)	+ 2.38 / - 0.45
15% in 24 h	21-40 min	0.686	0.001	101.13	51.6 (45.5, 57.7)	85.5 (81.2, 89.8)	+ 3.56 / - 0.57
15% in 24 h	41-60 min	0.681	0.001	99.25	48.4 (42.3, 54.5)	86.0 (81.8, 90.2)	+ 3.46 / - 0.60
15% in 24 h	61-90 min	0.683	0.001	90.25	61.3 (55.4, 67.2)	71.9 (66.4, 77.4)	+ 2.18 / - 0.54
15% in 24 h	91-120 min	0.730	<0.0001	93.17	64.5 (58.6, 70.4)	77.8 (72.7, 82.9)	+ 2.91 / - 0.46

AUC – area under the curve, CI – confidence interval, h – hours

*Values next to the + sign, represent the likelihood ratio for positive results and those next to the – sign represents the likelihood ratio for negative results. This means that the positive results are associated with postpartum hemorrhage (PPH) and the negative results are associated with absence of PPH.

## DISCUSSION

Our study aimed to evaluate whether changes in vital signs of postpartum women were correlated with the percentage of body blood volume (BBVp) lost after vaginal delivery. Our findings show that among SI, HR, SBP, DBP, RR and SatO2, increased SI and HR values were positively associated with the 90^th^ percentile of BBVp lost after delivery with high specificity but low sensitivity.

The actual diagnosis of PPH and severe PPH is defined as blood loss above 500 or 1000 mL after delivery, respectively [6]. However, we believe that the impact of losing more than 500 mL of blood differs depending on the woman’s initial BBV, which depends on their BMI. Changes in the cardiovascular system during pregnancy may protect women from dying due to hemorrhage. These changes start around the sixth week of pregnancy with a 45% increase in blood volume (1200-1600 mL), reaching a maximum volume of 4700-5200 mL at around 32 weeks [16,17].

This means that for a woman with a normal BMI at term, losing 1000 mL may represent less than 20% of her BBV, which could be well tolerated; however, if the woman has a BMI below 19, and consequently a lower BBV, losing 1000 mL may represent more than 30% of her BBV, which could be a life-threatening condition. In our sample, 38% of women in the < p90 group bled more than 500 mL within 24 hours of birth with no clinical repercussion. If the WHO definition of PPH were used to start treatment, more than a third (87/260) would receive unnecessary treatment. Therefore, individualizing the amount of blood loss could help us to improve diagnostic accuracy. Here, we show a distribution of BBVp ranging from 1% to 15%. To the best of our knowledge, this is the first study to objectively evaluate postpartum blood loss and compare it with individual BBVp loss. This approach is innovative and should be explored in further research as it considers an individualized parameter for blood loss, not a single cut-off point.

Similarly, the evaluation of clinical signs should also be individualized. In the majority of healthy pregnant and postpartum women, physiological compensatory mechanisms inhibit decreases in blood pressure and increases in heart rate until a large amount of blood has been lost (usually more than 1500 mL) [17,18]. Hence, changes in vital signs resulting from hemorrhage appear late in the process and may not assist in the early identification of PPH, despite being recommended by many clinicians and researchers [19].

Our results for SI and HR show high specificity but low sensitivity, with a slightly better performance for SI at all times when the likelihood ratio was evaluated. As the majority of blood loss occurs within 2 hours of delivery, we looked for changes in vital signs during the first 40 minutes that could help the identification of BBVp in the 90^th^ percentile in order to diagnose PPH early. Nevertheless, the low sensitivity of SI and HR suggests that these vital signs are limited in their ability to quickly identify increased bleeding. However, both SI and HR show high specificity, and values below the defined cut-off points could be used to rule out increased vaginal bleeding.

Women who bled above the 90^th^ percentile in the first 2 hours had a higher velocity of bleeding. Therefore, these women were at higher risk of an adverse outcome due to PPH, and for this group, an SI value above 1.04 and HR above 105.4 bpm presented the highest specificity for alerting medical practitioners to increased postpartum bleeding. On the other hand, if the women present SI below 1.04 and HR below 105.4 bpm within the first hours after delivery, the risk of them developing PPH is low.

Increases in SI values have been related to severe effects of PPH [20-24]. Sohn et al. showed that women with SI values above 0.9 have a 23% chance of receiving a massive transfusion (defined as the transfusion of more than 10 blood units) [23]. Similar results were found by Le Bas, who found that a mean SI value of 0.9 at 30 minutes after delivery was associated with massive PPH (defined by the authors as an estimated blood loss above 30% of BBV) [20].

Other studies have shown that SI values above 0.9 are associated with severe maternal outcomes due to PPH, including maternal death, ICU admission, blood transfusion of ≥4 blood units and invasive surgical interventions.(21,24) A previous study from our group showed that 30 minutes after delivery, the mean SI value found among women who received blood transfusion after vaginal delivery due to PPH was 0.88 ± 0.26 [22].

A study that evaluated the normal ranges of SI within the first hour after delivery showed that the mean SI value during this period was 0.66, and the upper quartile was 0.74 [25]. Our study showed that SI values above 1.04 could alert physicians to a percentage of BBV loss above the 90^th^ percentile before cardiovascular decompensation. Nonetheless, an easier to apply cut-off value in daily practice is an SI higher than 1.0 within 2 hours after birth, which means the HR is higher than the SBP. Conversely, when the HR value is below the SBP, the chance of developing PPH is low.

These findings have special applicability in low- and middle-income countries, as health providers could use the SI and HR as tools to identify which patients require a referral for further treatment, as well as those who are at low risk for developing PPH. Women delivering at home or in a low-level facility have a higher risk of PPH (as the frequency of active management of the third phase of labor is minimal) and a higher risk of death. Vital signs can be used as an alert for the health provider who is assisting the woman at home, or this could be taught to a family member. Delays in global health are a real issue. Vital signs can be useful to decrease the first and second delays, mainly in the case of home births or when childbirth is occurring in a lower-level facility. The delay in identifying PPH (first delay) and in deciding to transfer the patient to a high-level facility (second delay) can potentially be avoided when an accessible and simple tool can be used in home births or at a low-level facility to help the health provider or an informed family member begin treatment when there is suspicion of PPH.

Aside from this, secondary prevention for PPH using misoprostol has been discussed in countries where accessibility to a health facility is difficult or expensive [26]. Only women with increased values of SI and/or HR could receive this secondary prevention rather than universal management, which can reduce costs and the side effects of this medication.

We understand that SI and HR are not perfect screening tools, as both presented low sensitivity. However, it can be used with high specificity to identify women who should be more closely observed. Furthermore, assessing pros and cons in each situation, the health provider can start treatment (even if the blood is not yet exteriorized) or prepare for transfer.

Our study presents some limitations, and the findings should be interpreted in the context of these limitations. The sample size did not have sufficient power to identify severe maternal outcomes due to PPH as we had only few cases of blood transfusion and no cases requiring ICU admission. Therefore, we cannot correlate the loss of ≥2 mg/dL of Hb or even the 90^th^ BBVp percentile to a severe maternal outcome. Additionally, the BBV was calculated based on the BMI rather than the fat and lean body composition percentage, which could show different results. On the other hand, this study provides further insight into the physiology of postpartum women, and helps to identify those women that should receive more attention. Further research should aim to clarify the role of these results in the identification of severe maternal outcomes due to PPH, and to develop a standard tool that could help individualize the diagnosis of PPH.

## CONCLUSION

Use of SI and HR for the early identification of PPH presented high specificity but low specificity. However, SI values above 1.04 and HR above 105 bpm could be used as an alert to identify women who may experience blood loss above the 90^th^ percentile after childbirth. Use of the percentage of body blood volume lost after delivery could be a strategy to improve PPH diagnosis at an individual level, and this requires further discussion.
